# Comment on "A comprehensive overview and evaluation of circular RNA detection tools"

**DOI:** 10.1371/journal.pcbi.1006158

**Published:** 2019-05-31

**Authors:** Chia-Ying Chen, Trees-Juen Chuang

**Affiliations:** Genomics Research Center, Academia Sinica, Taipei, Taiwan; University of Canterbury, NEW ZEALAND

A recent paper published in *PLOS Computational Biology* [1] provided a comprehensive evaluation of various circular RNA (circRNA)-detection tools. The authors compared 11 different circRNA-detection tools using four different datasets, including three simulated datasets (positive, background, and mixed datasets) and one real dataset. Since the advent of high-throughput next-generation sequencing technology, dozens of computational tools have been developed and used to successfully detect thousands of circRNAs in a diverse range of species. However, there are great discrepancies in the results obtained using different tools [2–7], and systematic evaluations of their performance have not been available. Indeed, the cited work has provided a useful guideline for researchers engaged in circRNA studies. Nevertheless, it seems inappropriate to use all CircBase-deposited circRNA candidates (14,689 events) identified *in silico* from RNA-seq data of HeLa cells [8] as the positive dataset. The qualification of the 14,689 candidates requires further evaluation. We suggest that three main confounding factors, which may affect the fairness of the evaluation of circRNA-detection tools, should be considered.

First, it has been shown that non-co-linear (NCL) junctions (including circRNA and *trans*-spliced RNA junctions) that do not match annotated exon boundaries tend to be unreliable and are more likely to stem from mis-splicing [9–12], although we cannot eliminate the possibility that a few true backspliced junctions indeed originate from unannotated gene loci. Since circRNA candidates are regarded to be less or more reliable if their normalized read counts are depleted or enriched after RNase R treatment, respectively [13], we reexamined the circRNA candidates detected on the HeLa RNase R-treated and untreated samples (the circRNA candidates and the corresponding read counts were downloaded from the cited study). Of the circRNA candidates with unannotated exon boundaries, we can find that 50%~100% of them were “completely” depleted (not detected) after RNase R treatment, whereas only <8% of them were “significantly” enriched (i.e., 5-fold increase in normalized read count) after RNase R treatment (Fig 1). This result revealed that the candidates with unannotated exon boundaries are more likely to be false calls. Thus, we suggest that the CircBase circRNA candidates with unannotated exon boundaries (1,046 events; Table 1) should be excluded from the positive dataset. At least, since circRNA junctions were observed to be predominantly located at canonical splice sites [14–16], the candidates with junctions that have not canonical splice site sequences (GT-AG, GC-AG, or AT-AC) should be removed (778 events; Table 1).

**Fig 1:**
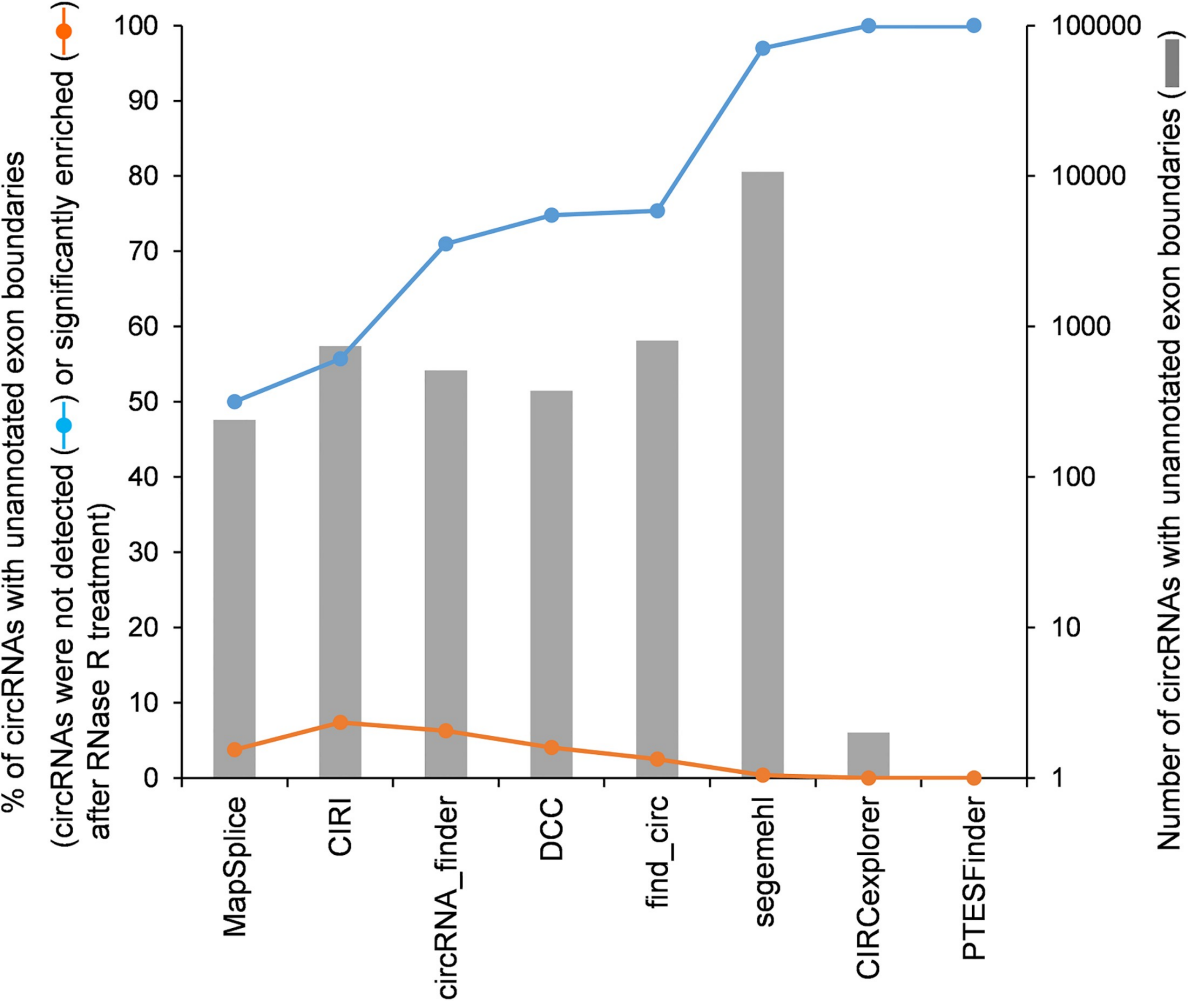
Analysis of the circRNA candidates that have unannotated exon boundaries and were not detected or significantly enriched (≥5 folds of enrichment in normalized read counts) after RNase R treatment on the HeLa samples. Only the circRNAs with ≥2 supporting NCL junction reads without RNase R treatment were considered. Of note, no circRNAs with unannotated exon boundaries were detected by UROBORUS, KNIFE, and NCLscan on the HeLa samples without RNase R treatment.

**Table 1:** Uncertain circRNA candidates in CircBase (see also S1 Dataset).

Confounding factors	Number of circRNAs
Junctions not matching annotated exon boundaries (A)	1,046
Non-canonical splice site^1^	778
Ambiguous alignment (B)^2^	2,316
Alternative co-linear explanation	514
Multiple hits	1,802
**Total uncertain circRNA candidates((A)∪(B))**	**3,110**

^1^The backspliced junctions have not canonical splice site sequences (GT-AG, GC-AG, or AT-AC).

^2^The extracted concatenated sequences were aligned against the reference/annotated transcripts using BLAT with two parameter sets: (-titleSize = 9, -stepSize = 9, -repMatch = 32768; default parameters of the Windows version) and (-titleSize = 11, -stepSize = 11, -repMatch = 1024; default parameters of the stand-alone version). A concatenated sequence is regarded as an “alternative co-linear explanation” if its BLAT-alignment results contain at least one co-linear explanation.

Second, ambiguous alignments originating from repetitive sequences or paralogous genes often result in false positive circRNA detection. In CircBase, most circRNA candidates were identified by find_circ [8]. It has been reported that some of find_circ-identified candidates were mis-predicted from paralogous genes [17]. Therefore, the factor of alignment ambiguity should be considered when using CircBase circRNAs as true positives. To this end, we concatenated the exonic sequence flanking the circRNA junction (within -100 nucleotides to +100 nucleotides of each CircBase-identified junction; see Fig 2A). We then aligned the 200 bp concatenated sequence (the concatenated sequence may be shorter than 200 bp if the exonic circRNA sequence is shorter than 200 bp) against the reference genome and NCBI RefSeq-/GENCODE-identified mRNAs using BLAT [18]. It is worth noting that 2,316 concatenated sequences exhibited ambiguous alignments (Table 1), of which 514 events contained alternative co-linear explanations (an example was illustrated in Fig 2B) and 1,802 events mapped to multiple positions with similar BLAT mapping scores (difference of mapping scores < 5) (an example was illustrated in Fig 2C). These 2,316 CircBase circRNAs are not appropriate for inclusion in the positive dataset. We further examined the 2,316 potentially false calls and found that they contributed to 4%~14% of the identifications among the 11 tools (Fig 2D). Although these false calls can be considerably eliminated by merging two circRNA predicting tools, the filtering performance is dependent on tools used (Fig 2E). These results also suggested that these false calls were not the find_circ specific issue in CircBase.

**Fig 2:**
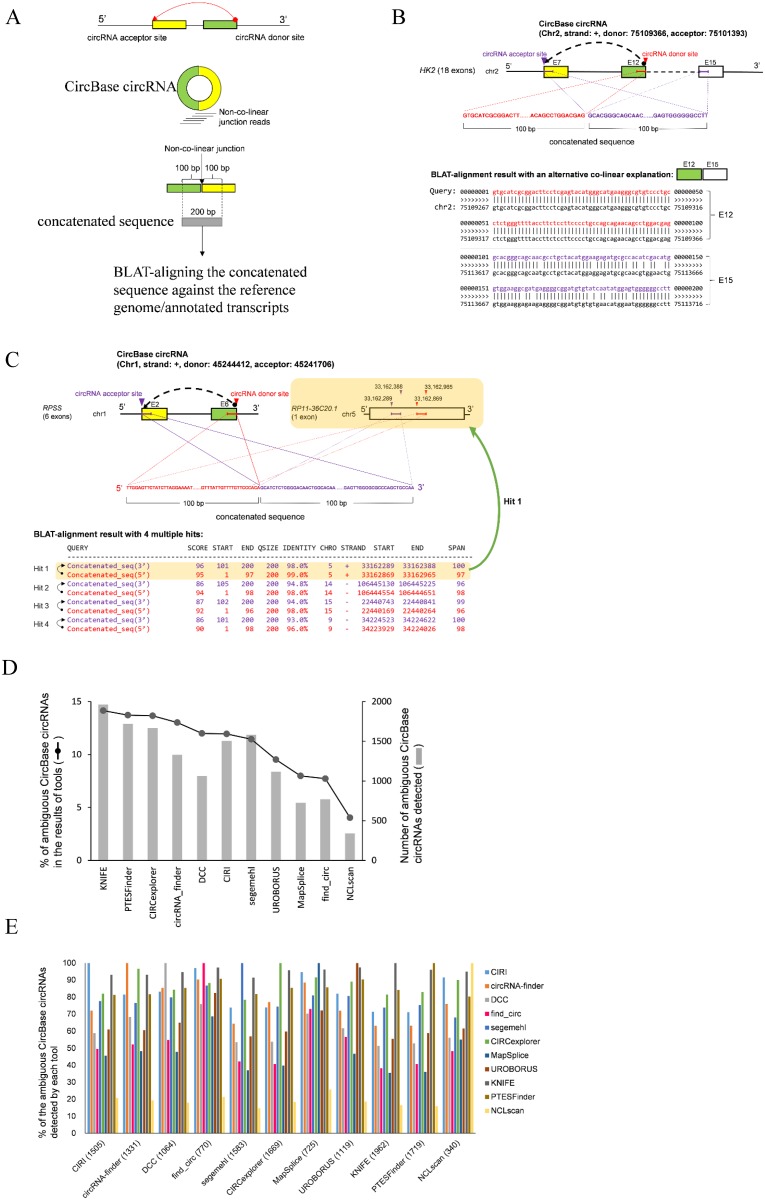
Detection of CircBase circRNAs with ambiguous alignments. (A) Schematic illustration of a concatenated sequence derived from a CircBase circRNA. (B and C) Examples of CircBase circRNAs with an alternative co-linear explanation (B) and multiple hits (C). For (B), the concatenated sequence of the CircBase circRNA (E12-E7) had an alternative co-linear explanation (E12-E15). For (C), the concatenated sequence of the CircBase circRNA (E6-E2) mapped to multiple positions (Hits 1–4). E, exon. (D) Distribution of the ambiguous CircBase circRNAs (2,316 events) in the results of the 11 tools (see also S2 Dataset). (E) Percentage of the ambiguous CircBase circRNA candidates of a tool when considering another circRNA predicting tool. The numbers in parentheses were ambiguous CircBase circRNA candidates detected by each tool.

Third, of the CircBase circRNAs, 3,580 events were identified by all 11 tools. We observed that the number of identified backspliced junction reads varied remarkably between tools depending on the level of strictness of the filtering steps used (Fig 3A and a similar result shown in Fig. 4C of the cited study [1]). With relatively strict filtering criteria, UROBORUS, NCLscan, circRNA_finder, and CIRCexplorer provided a higher percentage of identified circRNAs with one supporting junction read than did the other tools (Fig 3B). These four tools and DCC provided a relatively low percentage (<10%) of identified circRNAs with >10 supporting junction read, whereas such percentages were more than 60% for CIRI and MapSplice (Fig 3B). For example, for the CircBase circRNA candidate of *COQ4* (Fig 3C, top), CIRI identified five supporting junction reads, whereas UROBORUS and NCLscan identified one supporting junction read only (the information of supporting junction reads were extracted from the github website generated by the cited study at https://github.com/linatbeishan/circRNA_detection [1]). However, four of the five CIRI-provided junction reads were unqualified reads with ambiguous alignments of an alternative co-linear explanation (Fig 3C, middle and bottom), if realigned against the reference genome and annotated mRNAs. Such unqualified reads were not included in the UROBORUS and NCLscan results. To evaluate the impact of the unqualified reads on circRNA predictions, we took the result of CIRI identification as an example (because only CIRI provided the IDs of supporting backspliced junction reads). We found that as high as 50% (57,288 reads) of the CIRI-provided backspliced junction reads were unqualified (Fig 3D, left). After removing these unqualified reads, the number of the detected circRNA candidates (Fig 3D, left) and the median number of supporting backspliced junction reads per candidate (Fig 3D, middle) remarkably decreased; meanwhile, the percentage of the CIRI-identified circRNAs with one supporting junction read significantly increased (from 0% to 5%; Fig 3D, right). These results revealed that the unqualified reads would considerably affect the accuracy of circRNA predictions and should be carefully controlled. In addition, as also noted in the cited study [1], different tools use different counting methods to deal with a read pair spanning the same backspliced junction. Thus, it would be unfair to evaluate sensitivity among tools while directly considering candidates with ≥ 2 supporting junction reads (i.e., Table 1 of the cited study [1]). By the same reason, it is improper to assess sensitivity among tools at the read level without accounting for read qualification (i.e., Fig. 4 of the cited study [1]).

**Fig 3:**
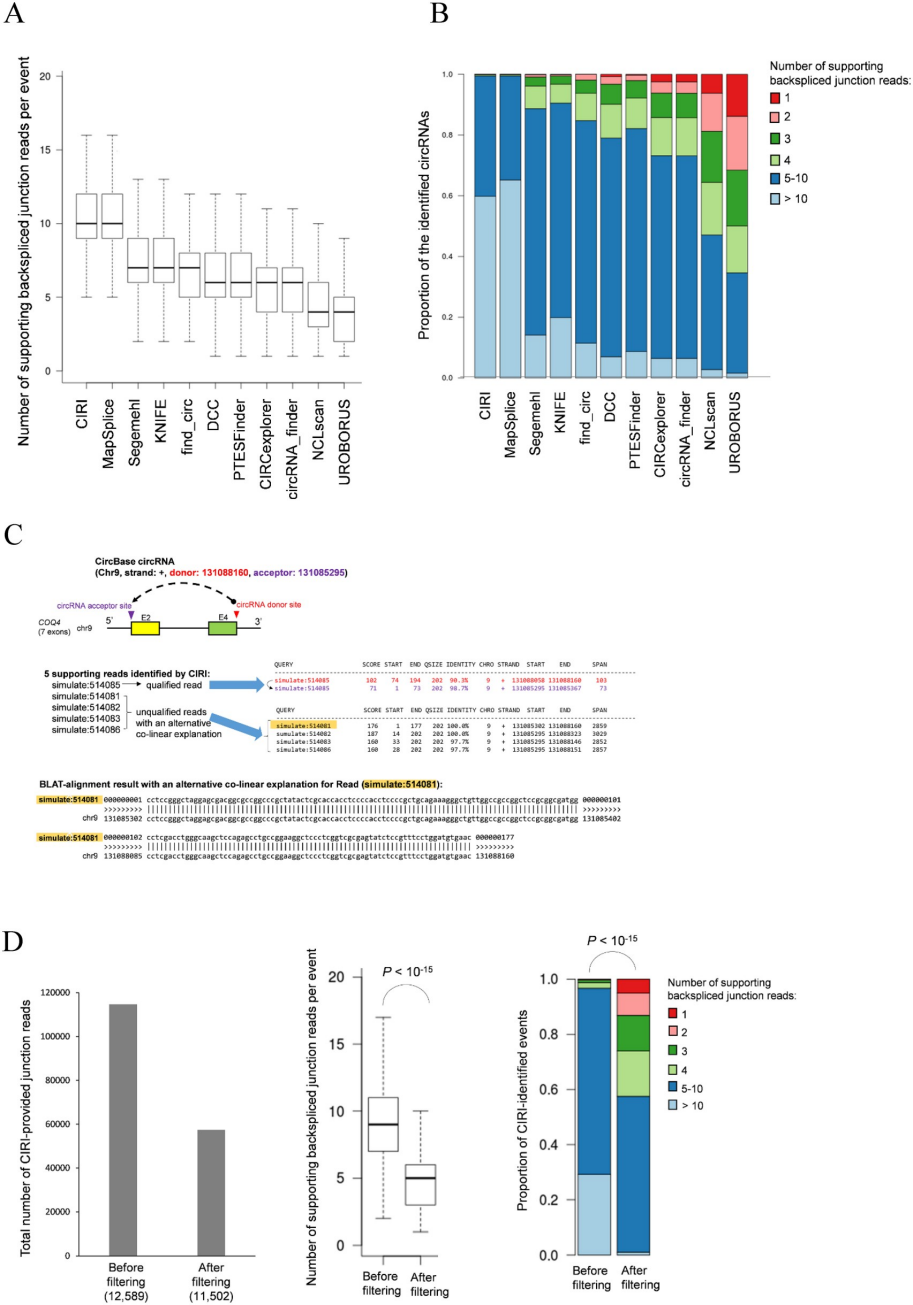
Variations in (A) number of identified junction reads among the 11 tools and (B) distribution of supporting junction reads for each tool. The 3,580 CircBase circRNAs identified by all 11 tools were considered. (C) Example of unqualified RNA-seq reads (i.e., ambiguous alignments with an alternative co-linear explanation or multiple hits). In this case (CircBase circRNA of *COQ4*, top), CIRI identified five supporting circRNA junction reads, four of which were unqualified reads with an alternative co-linear explanation. The BLAT-alignment result with an alternative co-linear explanation for Read (simulate:514081) was shown (bottom). E, exon. (D) Comparison of CIRI-identified circRNA candidates before and after removing the unqualified reads. For the left panel, the numbers of CIRI-identified circRNA candidates were provided in parentheses. For the middle and right panels, the *P* values were evaluated using the two-tailed Wilcoxon rank-sum and *Chi*-square (for given probabilities) tests, respectively.

Identification of NCL RNAs is often hampered by false positives arising from sequencing/alignment errors (particularly alignment ambiguity as stated above) and experimental artifacts (particularly *in vitro* artifacts arising from template switching during reverse transcription (RT)) [11, 12, 19–22]. Selection of positive/negative datasets (positive datasets particularly) for evaluating accuracy of circRNA-detection tools should be careful. Generally, positive datasets are generated or selected from: (1) simulation, (2) real RNA-seq datasets from samples with different treatments, and (3) wet-laboratory validated datasets. For simulated datasets, the co-linear (false positive or background) and circRNA (true positive) reads are generated from well-annotated co-linear mRNAs. Alternatively, in the cited study, the true positive reads were generated from *in silico* identified circRNA candidates (i.e., CircBase circRNAs) [1]. Several read simulators such as Mason [23], flux [24], ART [25], and CIRI-simulator [17] (used in this cited study) have been developed and successfully applied to generating reads. Since the authenticity of the synthetic datasets is totally unknown, the ambiguity/uncertainty of the simulated positive datasets should be minimized. We suggest that the simulated circRNA reads should not be generated from gene families, pseudogenes, mitochondrial/ribosomal genes, or unannotated exon boundaries (or junctions not having canonical splice site sequences). In addition, it would be better to evaluate accuracy of tools based on simulated data with a variety of data conditions of different read depths and of different read lengths, because accuracy of circRNA detection is often susceptible to these two factors [2, 7]. The main flaw of these simulation-based approaches is that they are harmful to assess the effect of experimental artifacts on tools. For real RNA-seq datasets, although comparisons of NCL events detected with and without RNase R-treatment [13] (or oligo-dT selection [26]) have been used to demarcate circRNAs versus non-circRNA NCL events, such approaches cannot distinguish between *in vivo* circRNAs and RT-based artifacts (e.g., template switching events). To control for experimental artifacts, an approach based on *Drosophila* hybrid mRNAs (*D*. *melanogaster* females vs. *D*. *sechellia* males) and a mixed mRNA-negative control sample was developed and successfully detected a considerable number of experimental artifacts [22]. However, it would be impossible to apply this approach to human studies. Recently, on the basis of the concept that RTase-dependent RNA products are likely to be RT artifacts [2, 11, 12, 27, 28] and comparisons of different RTase products could act as effectively as RTase-free validation when detecting RT-based artifacts [11, 12], we recently described an alternative pipeline to systematically extract potentially true positive circRNAs with controlling for experimental artifacts [29]. The RNA-seq reads were generated from Avian Myeloblastosis Virus (AMV)- and Moloney Murine Leukemia Virus (MMLV)-derived samples, respectively. Only the non-poly(A)-selected RNA products, in which their NCL junctions were supported by both AMV- and MMLV-based reads, were regarded as true positive circRNAs [29]. Finally, wet-laboratory validated circRNAs often serve as true positives and are employed for evaluating the sensitivity of tools. However, the collected circRNAs tested in various tissues or cell lines. It has been shown that most circRNAs were expressed in only a few tissues/cell types [17, 29–33]. As stated in the cited study [1], many of the collected circRNAs may not be expressed in the examined samples. In addition, we especially emphasize that the collected circRNAs should not include circRNAs validated by RT-PCR experiments with only one type of RTase because they may be RT-dependent RNA products and derived from *in vitro* artifacts [12]. To minimize potential RT-artifacts, the selected circRNAs should be confirmed by both RT- and non-RT-based experiments (e.g., Northern blot or RNase protection assay [34]) or at least, by different two types of RTase-based experiments [11, 12, 29].

With more and more circRNAs are detected, the reliability and function of most identified circRNAs remains an open question worthy of further investigation. To reduce the cost of further validation and functional analysis, it is necessary to carefully evaluate the reliability of the used circRNA-detecting tools. The abovementioned factors may considerably affect the evaluation results and partially explain the discrepancy observed in the sensitivity of the examined tools using synthetic (52–92%; Table 1 of the cited study [1]) and real (46–63% on HeLa cells; Fig. 5 of the cited study [1]) datasets. Therefore, we suggest that the accuracy of the 11 tools should be reevaluated (e.g., Table 1 and Fig. 4 of the cited study [1]), taking into account the factors discussed above when using the positive datasets.

## Data access

The publicly available toolkit for downstream filtering of circRNA predictions with ambiguous alignment is downloadable at https://github.com/TreesLab/NCLtk.

## Supporting information

S1 Dataset The 3,150 uncertain CircBase circRNAs (summarized in Table 1) and the 3,580 CircBase circRNAs identified by all the 11 tools examined (used in Fig 3A). (XLSX)

S2 Dataset Summary of the 2,316 ambiguous CircBase circRNAs in the results of the 11 tools. (XLSX)

## References

[1] Zeng X, Lin W, Guo M, Zou Q (2017) A comprehensive overview and evaluation of circular RNA detection tools. PLoS Comput Biol 13: e1005420 10.1371/journal.pcbi.1005420 28594838 PMC5466358

[2] Chen I, Chen CY, Chuang TJ (2015) Biogenesis, identification, and function of exonic circular RNAs. Wiley Interdiscip Rev RNA 6: 563–579. 10.1002/wrna.1294 26230526 PMC5042038

[3] Abate F, Acquaviva A, Paciello G, Foti C, Ficarra E, et al (2012) Bellerophontes: an RNA-Seq data analysis framework for chimeric transcripts discovery based on accurate fusion model. Bioinformatics 28: 2114–2121. 10.1093/bioinformatics/bts334 22711792

[4] Nacu S, Yuan W, Kan Z, Bhatt D, Rivers CS, et al (2011) Deep RNA sequencing analysis of readthrough gene fusions in human prostate adenocarcinoma and reference samples. BMC Med Genomics 4: 11 10.1186/1755-8794-4-11 21261984 PMC3041646

[5] Carrara M, Beccuti M, Cavallo F, Donatelli S, Lazzarato F, et al (2013) State of art fusion-finder algorithms are suitable to detect transcription-induced chimeras in normal tissues? BMC Bioinformatics 14 Suppl 7: S2. 10.1186/1471-2105-14-S7-S2 PMC3633050 23815381

[6] Carrara M, Beccuti M, Lazzarato F, Cavallo F, Cordero F, et al (2013) State-of-the-art fusion-finder algorithms sensitivity and specificity. Biomed Res Int 2013: 340620 10.1155/2013/340620 23555082 PMC3595110

[7] Chuang TJ, Wu CS, Chen CY, Hung LY, Chiang TW, et al (2016) NCLscan: accurate identification of non-co-linear transcripts (fusion, trans-splicing and circular RNA) with a good balance between sensitivity and precision. Nucleic Acids Res 44: e29 10.1093/nar/gkv1013 26442529 PMC4756807

[8] Glazar P, Papavasileiou P, Rajewsky N (2014) circBase: a database for circular RNAs. RNA 20: 1666–1670. 10.1261/rna.043687.113 25234927 PMC4201819

[9] Kim P, Yoon S, Kim N, Lee S, Ko M, et al (2010) ChimerDB 2.0—a knowledgebase for fusion genes updated. Nucleic Acids Res 38: D81–85. 10.1093/nar/gkp982 19906715 PMC2808913

[10] Al-Balool HH, Weber D, Liu Y, Wade M, Guleria K, et al (2011) Post-transcriptional exon shuffling events in humans can be evolutionarily conserved and abundant. Genome Res 21: 1788–1799. 10.1101/gr.116442.110 21948523 PMC3205564

[11] Wu CS, Yu CY, Chuang CY, Hsiao M, Kao CF, et al (2014) Integrative transcriptome sequencing identifies trans-splicing events with important roles in human embryonic stem cell pluripotency. Genome Res 24: 25–36. 10.1101/gr.159483.113 24131564 PMC3875859

[12] Yu CY, Liu HJ, Hung LY, Kuo HC, Chuang TJ (2014) Is an observed non-co-linear RNA product spliced in trans, in cis or just in vitro? Nucleic Acids Res 42: 9410–9423. 10.1093/nar/gku643 25053845 PMC4132752

[13] Hansen TB, Veno MT, Damgaard CK, Kjems J (2016) Comparison of circular RNA prediction tools. Nucleic Acids Res 44: e58 10.1093/nar/gkv1458 26657634 PMC4824091

[14] Szabo L, Morey R, Palpant NJ, Wang PL, Afari N, et al (2015) Statistically based splicing detection reveals neural enrichment and tissue-specific induction of circular RNA during human fetal development. Genome Biol 16: 126 10.1186/s13059-015-0690-5 26076956 PMC4506483

[15] Jeck WR, Sorrentino JA, Wang K, Slevin MK, Burd CE, et al (2013) Circular RNAs are abundant, conserved, and associated with ALU repeats. RNA 19: 141–157. 10.1261/rna.035667.112 23249747 PMC3543092

[16] Guo JU, Agarwal V, Guo H, Bartel DP (2014) Expanded identification and characterization of mammalian circular RNAs. Genome Biol 15: 409 10.1186/s13059-014-0409-z 25070500 PMC4165365

[17] Gao Y, Wang J, Zhao F (2015) CIRI: an efficient and unbiased algorithm for de novo circular RNA identification. Genome Biol 16: 4 10.1186/s13059-014-0571-3 25583365 PMC4316645

[18] Kent WJ (2002) BLAT—the BLAST-like alignment tool. Genome Research 12: 656–664. 10.1101/gr.229202 11932250 PMC187518

[19] Maher CA, Kumar-Sinha C, Cao X, Kalyana-Sundaram S, Han B, et al (2009) Transcriptome sequencing to detect gene fusions in cancer. Nature 458: 97–101. 10.1038/nature07638 19136943 PMC2725402

[20] Shao X, Shepelev V, Fedorov A (2006) Bioinformatic analysis of exon repetition, exon scrambling and trans-splicing in humans. Bioinformatics 22: 692–698. 10.1093/bioinformatics/bti795 16308355

[21] Ozsolak F, Milos PM (2011) RNA sequencing: advances, challenges and opportunities. Nat Rev Genet 12: 87–98. 10.1038/nrg2934 21191423 PMC3031867

[22] McManus CJ, Duff MO, Eipper-Mains J, Graveley BR (2010) Global analysis of trans-splicing in Drosophila. Proc Natl Acad Sci U S A 107: 12975–12979. 10.1073/pnas.1007586107 20615941 PMC2919919

[23] Holtgrewe M, Emde AK, Weese D, Reinert K (2011) A novel and well-defined benchmarking method for second generation read mapping. BMC Bioinformatics 12: 210 10.1186/1471-2105-12-210 21615913 PMC3128034

[24] Griebel T, Zacher B, Ribeca P, Raineri E, Lacroix V, et al (2012) Modelling and simulating generic RNA-Seq experiments with the flux simulator. Nucleic Acids Res 40: 10073–10083. 10.1093/nar/gks666 22962361 PMC3488205

[25] Huang W, Li L, Myers JR, Marth GT (2012) ART: a next-generation sequencing read simulator. Bioinformatics 28: 593–594. 10.1093/bioinformatics/btr708 22199392 PMC3278762

[26] You X, Conrad TO (2016) Acfs: accurate circRNA identification and quantification from RNA-Seq data. Sci Rep 6: 38820 10.1038/srep38820 27929140 PMC5144000

[27] Houseley J, Tollervey D (2010) Apparent non-canonical trans-splicing is generated by reverse transcriptase in vitro. PLoS One 5: e12271 10.1371/journal.pone.0012271 20805885 PMC2923612

[28] Kong Y, Zhou H, Yu Y, Chen L, Hao P, et al (2015) The evolutionary landscape of intergenic trans-splicing events in insects. Nat Commun 6: 8734 10.1038/ncomms9734 26521696 PMC4667647

[29] Trees-Juen Chuang Y-JC, Chen Chia-Ying, Mai Te-Lun, Wang Yi-Da, Yeh Chung-Shu, Yang Min-Yu, Hsiao Yu-Ting, Chang Tien-Hsien, Kuo Tzu-Chien, Cho Hsin-Hua, Shen Chia-Ning, Kuo Hung-Chih, Lu Mei-Yeh, Chen Yi-Hua, Hsieh Shan-Chi, and Chiang Tai-Wei (2018) Integrative transcriptome sequencing reveals extensive alternative trans-splicing and cis-backsplicing in human cells. Nucleic Acids Res 46: 3671–3691. 10.1093/nar/gky032 29385530 PMC6283421

[30] Salzman J, Chen RE, Olsen MN, Wang PL, Brown PO (2013) Cell-type specific features of circular RNA expression. PLoS Genet 9: e1003777 10.1371/journal.pgen.1003777 24039610 PMC3764148

[31] Memczak S, Jens M, Elefsinioti A, Torti F, Krueger J, et al (2013) Circular RNAs are a large class of animal RNAs with regulatory potency. Nature 495: 333–338. 10.1038/nature11928 23446348

[32] Westholm Jakub O, Miura P, Olson S, Shenker S, Joseph B, et al (2014) Genome-wide Analysis of Drosophila Circular RNAs Reveals Their Structural and Sequence Properties and Age-Dependent Neural Accumulation. Cell Reports 9: 1966–1980. 10.1016/j.celrep.2014.10.062 25544350 PMC4279448

[33] Starke S, Jost I, Rossbach O, Schneider T, Schreiner S, et al (2015) Exon Circularization Requires Canonical Splice Signals. Cell Rep 10: 1–9. 25543144 10.1016/j.celrep.2014.12.002

[34] Djebali S, Lagarde J, Kapranov P, Lacroix V, Borel C, et al (2012) Evidence for transcript networks composed of chimeric RNAs in human cells. PLoS ONE 7: e28213 10.1371/journal.pone.0028213 22238572 PMC3251577

